# Supplementary material for: Direct observation of the on-site oxygen 2p two-hole Coulomb energy in La2CuO4
Source: Nat Commun. 2025 Nov 5;16:9748. doi: 10.1038/s41467-025-65314-w (PMC12589636; doi:10.1038/s41467-025-65314-w)
Supplement: Supplementary file 1 — Supplementary Information [file 41467_2025_65314_MOESM1_ESM.pdf]

Supplementary Information

Direct Observation of the On-site Oxygen $2p$ Two-Hole Coulomb Energy in La_2CuO_4

Danilo Kühn[1,2*], Swarnshikha Sinha[1,2,3], Fredrik O. L. Johansson[2,4], Katarzyna Siewierska[1], Antonello Tebano[5,6], Nils Mårtensson[2,4], Andreas Lindblad[2,4], Daniele Di Castro[5,6], Alexander Föhlisch[1,2,3*]

[1*]Institut für Methoden und Instrumentierung der Forschung mit Synchrotronstrahlung, Helmholtz-Zentrum Berlin für Materialien und Energie GmbH, Albert-Einstein-Str. 15, Berlin, 12489, Germany.
[2*]Uppsala-Berlin Joint Laboratory on Next Generation Photoelectron Spectroscopy, Albert-Einstein-Str. 15, Berlin, 12489, Germany.
[3*]Institut für Physik und Astronomie, Universität Potsdam, Karl-Liebknecht-Straße 24/25, Potsdam, 14476, Germany.
[4]Department of Physics and Astronomy, Division of X-ray Photon Science, Uppsala University, P. O. Box 516, Uppsala, SE-752 37, Sweden.
[5]Dipartimento di Ingegneria Civile e Ingegneria Informatica, Università di Roma Tor Vergata, Via del Politecnico 1, Roma, 00133, Italia.
[6]CNR-SPIN, Università di Roma Tor Vergata, Roma, Italia.

*Corresponding author(s). E-mail(s): danilo.kuehn@helmholtz-berlin.de; alexander.foehlisch@helmholtz-berlin.de;

Supp. Note 1. Depth Probing by APECS

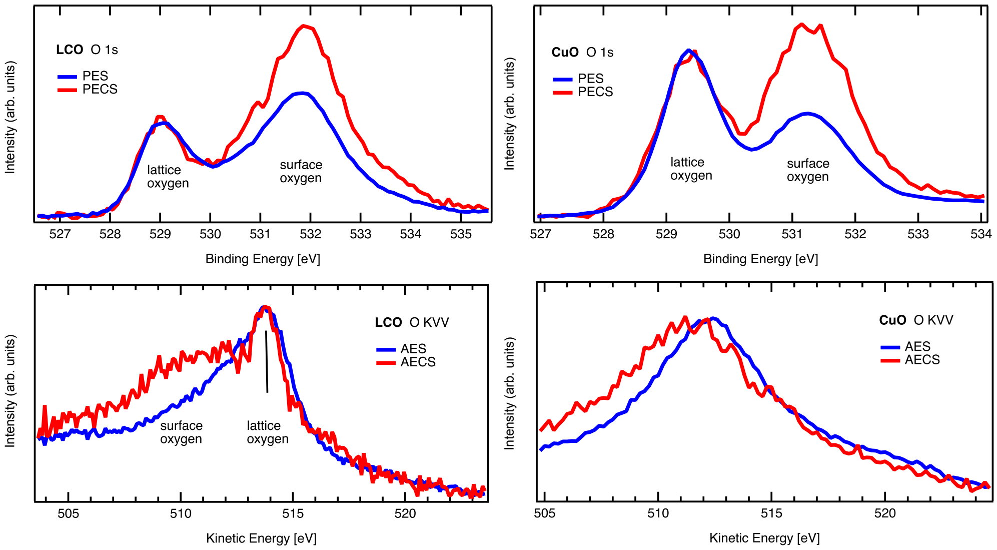

Supplementary Figure 1 Comparison of highly surface sensitive APECS measurements with deeper probing non-coincidence sub data sets. The AECS and PECS spectra of LCO and CuO are obtained from integration of the APECS maps in Fig. 2a,d over the full photoemission energy range (to obtain AECS) and the full Auger energy range (to obtain PECS), respectively. The PES and PECS spectra are normalized to the peak at about 529 eV binding energy, which we attribute to lattice oxygen. The second peak at about 532 eV (531.5 eV for CuO) is substantially increased in the highly surface sensitive coincidence measurement, validating our assignment of this feature to an oxygen species in the surface layer. In the AES of LCO, the intensity of the broad feature centered at 510 eV kinetic energy is increased in AECS, which gives further proof that it arises from the surface layer, whereas the peak at 514 eV can be attributed to lattice oxygen from the bulk. For CuO, it is difficult to assign the surface and bulk features directly in the AES. A constant background is subtracted from (non-coincidence) AES and PES to remove the inelastic background from unrelated features at higher kinetic energies.

The Mean Escape Depths (MED) of the electrons in LCO calculated with the TPP-2M formula[1] for emission towards the central axis of the spectrometers are: 0.22 nm for O 1s / O KVV coincidence electron pairs; 0.30 nm for O 1s (non-coincidence); 0.79 nm for O KVV (non-coincidence). The effective probing depth is an average of the MED's weighted over all take-off angles accepted by the spectrometers. The MED for coincidence electron pairs varies between 0.05 to 0.5 nm due to the large opening angles of the spectrometers. Also the non-coincidence MED varies with emission angle and is always higher than the corresponding coincidence one.[2, 3]. Source data are provided as a Source Data file.

Supp. Note 2. Additional Cini-Sawatzky Simulations

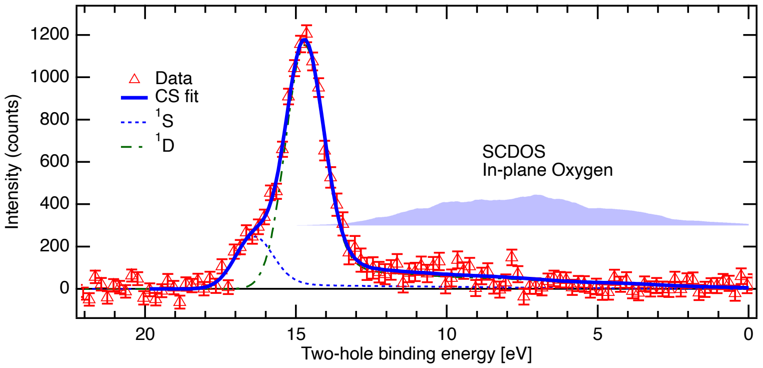

Supplementary Figure 2 O 2p two-hole spectrum of LCO measured over an extended energy range. The Auger spectrum was recorded with the 'Ang50_7pc' lens mode. The energy window size is increased and the energy resolution is decreased (about 1 eV), as compared to 'Ang56_4pc' mode (see Methods for details). The self-convoluted density of states (SCDOS's) based on the calculated PDOS's by Pickett et al. [4] is shown vertically offset. Data points with error bars are red, blue line shows the Cini-Sawatzky (CS) fit with individual $_1$S and $_1$D components (dashed lines). Vertical error bars are standard deviation, see Methods section. Source data are provided as a Source Data file.

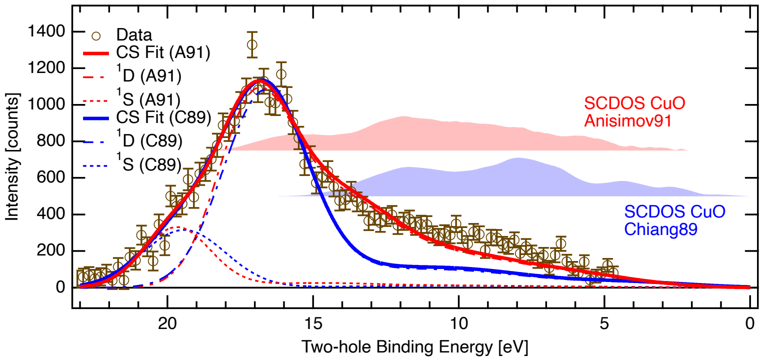

Supplementary Figure 3 Cini-Sawatzky fits of CuO with PDOS from Ching et al. and Anisimov et al. The self-convoluted density of states (SCDOS's) based on the calculated PDOS's from Ching et al. [5] (C89) and Anisimov et al. [6] (A91) are shown vertically offset. Data points with error bars are brown, blue and red lines show Cini-Sawatzky (CS) fits with individual ^{1}S and ^{1}D fit components (dashed lines). Vertical error bars are standard deviation, see Methods section. Source data are provided as a Source Data file.

Supp. Note 3. Partial Density of States for Cini-Sawatzky Simulations

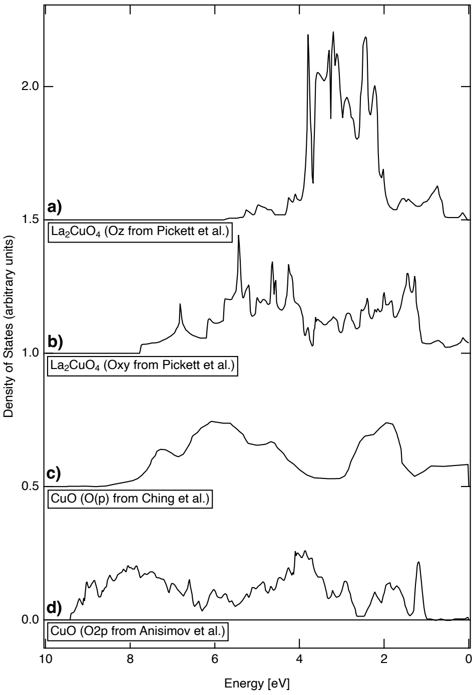

Supplementary Figure 4 Digitized O 2p PDOS: The PDOS's were digitized from: **a,b** Pickett et al.[4] Fig. 1 left Oz (**a**) and Oxy (**b**), **c** Ching et al.[5] Fig. 7 O(p) and **d** Anisimov et al.[6] Fig. 4 O 2p. The PDOS are normalized to area. It is not the intention to reproduce the calculated PDOS shapes in all detail. However, the overall energy position of the PDOS's have been tried to be reproduced as accurate as possible.

Supp. Info 4. X-ray diffraction, Reciprocal Space Mapping and X-ray Reflectivity Sample Characterizations

The structural quality of the LCO films was characterized using X-ray diffraction (XRD), reciprocal space mapping (RSM) and X-ray reflectivity (XRR). The symmetric XRD scan in Supp. Fig. 5(a) confirms single phase epitaxial growth of LCO on the SLAO(001) substrate. Well evident finite size oscillations and narrow (FWHM $< 0.1°$) rocking curve, shown in Supp. Figs. 5(b) and 5(c), respectively, indicate uniform film thickness (about 40 nm), smooth interfaces and high crystalline quality, with low density of extended defects. The reciprocal space map (RSM) in Supp. Fig. 6, measured around the SLAO (107) reflection, enables the determination of both in-plane and out-of-plane lattice parameters of the LCO film. While the film likely experiences substrate-induced strain near the interface, its thickness allows for strain relaxation. The measured lattice parameters, $a = 81 \pm 1$ pm and $c = 1312 \pm 4$ pm, indicate that the film has relaxed toward its bulk structure. XRR measurements (not shown) were done on a second LCO sample grown with the identical setup under same conditions. The XRR measurements indicate a surface roughness of approximately 2 nm.

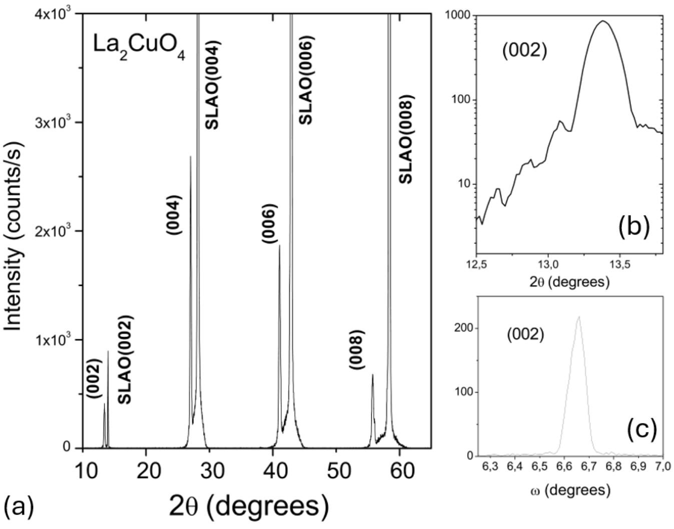

Supplementary Figure 5 (a) Symmetric XRD scan of the LCO film on SLAO substrate with labelled reflections substrate pattern, (b) the finite size oscillations and (c) the rocking curve of the (002) peak. Source data are provided as a Source Data file.

To characterize the structure of the film grown on Cu, we performed X-ray diffraction (XRD) measurements. Symmetric XRD scans using a parallel beam configuration ($\Theta = \omega$) primarily probe lattice planes that are parallel to the sample surface, making this geometry well-suited for confirming the orientation of single crystals such as the underlying Cu (110). However, this configuration is less sensitive to misoriented or

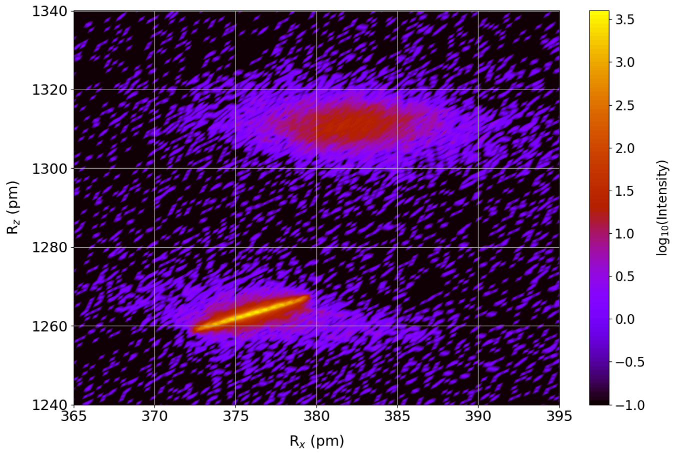

Supplementary Figure 6 Reciprocal space map of a 56 nm LCO thin film on SLAO substrate performed around the (107) reflection confirming the relaxation of the thin film with lattice parameters a = 381 ± 1 pm and c = 1312 ± 4 pm. Source data are provided as a Source Data file.

polycrystalline phases. To enhance the visibility of diffraction peaks from the oxidized overlayer, which may exhibit various orientations, we offset the incidence angle ω by a small amount (e.g., 1°). This allows detection of off-normal planes and improves sensitivity to non-epitaxial phases. The symmetric XRD scan in Supp. Fig. 7 confirms the (110) orientation of the Cu single crystal substrate. To identify the oxide phases and assign the observed diffraction peaks, we analysed the data collected with the 1° ω-offset scan. The measured XRD pattern, shown in Supp. Fig. 8, was compared to reference patterns of cubic Cu_2O, monoclinic CuO, and orthorhombic CuO_2. No peaks corresponding to orthorhombic CuO_2 were observed. All visible reflections can be attributed to cubic Cu_2O and monoclinic CuO. The data indicate that the Cu_2O layer formed on the Cu(110) single crystal is polycrystalline and exhibits some degree of texturing. The overlying CuO layer is also polycrystalline; however, a pronounced (111) texture appears to dominate, likely due to the lower surface energy associated with this orientation. Analysis of the CuO(111) reflection using the Scherrer equation yields a structural coherence length of approximately 17 ± 2 nm along the [111] direction. This value represents the average distance over which the CuO lattice remains well-ordered and free from significant disruptions such as stacking faults, dislocations, or grain boundary misorientations. It serves as a lower bound for the actual grain size. In the literature, CuO films formed by oxidation of Cu_2O exhibit grain sizes ranging from 20 to 120 nm, depending on oxidation conditions [7]. The structural coherence length estimated in our study ($\approx 17 \pm 2$ nm) is consistent with the lower end of this range, indicating the presence of well-ordered crystalline domains of comparable scale.

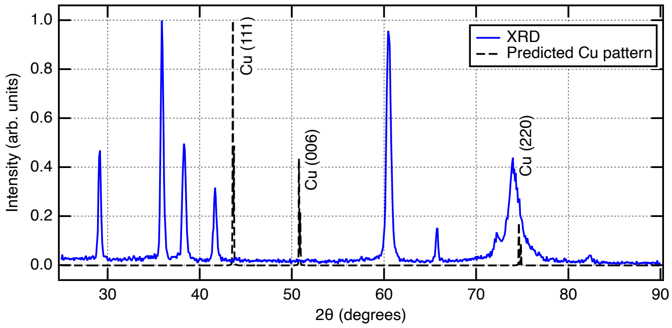

Supplementary Figure 7 Symmetric XRD scan (parallel beam geometry) collected on the oxidized Cu sample. A prominent peak corresponding to the Cu (220) reflection is observed, while the Cu (111) and Cu (006) reflections are absent, confirming the (110) orientation of the Cu single crystal substrate. Additional peaks correspond to the oxide overlayer. Source data are provided as a Source Data file.

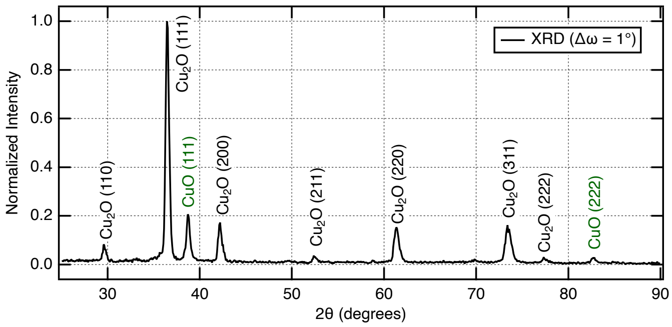

Supplementary Figure 8 XRD scan collected with an ω offset of 1° ($\Delta\omega = 1°$) on the oxidized Cu sample. The Cu (220) reflection is suppressed due to the offset geometry. All visible reflections can be assigned to cubic Cu_2O and monoclinic CuO phases. Source data are provided as a Source Data file.

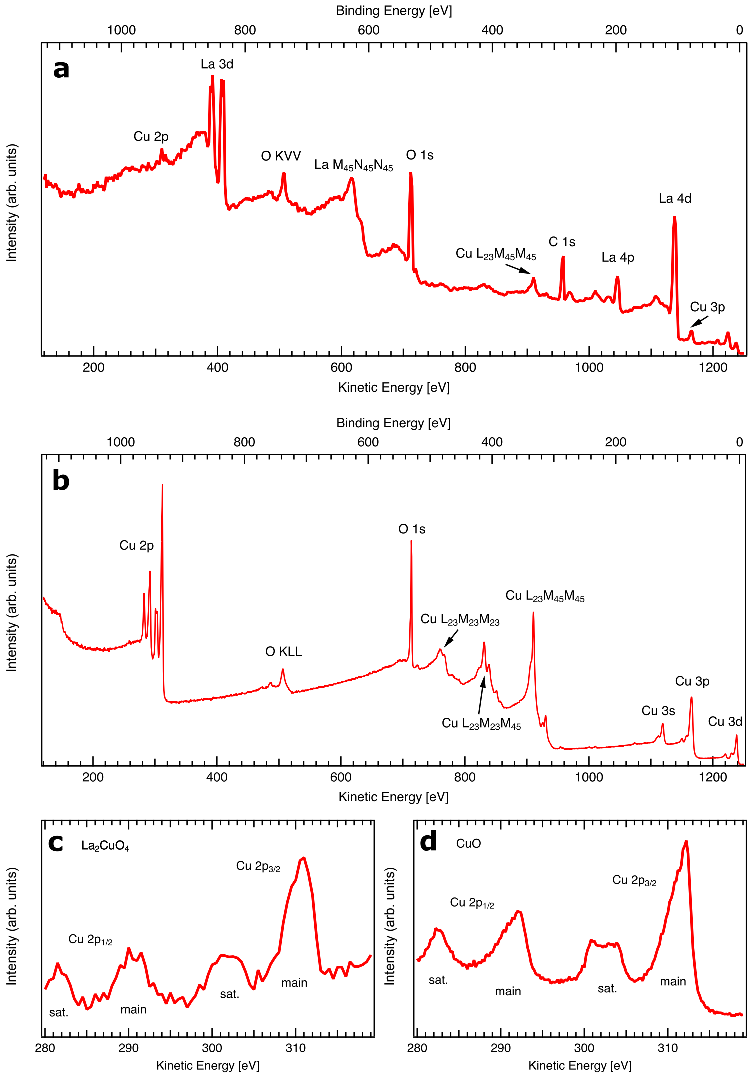

Supplementary Figure 9 XPS measurements of LCO and CuO. Survey XPS of LCO (**a**) and of CuO (**b**), measured at E_{ph}=1250 eV. The spectra show copper and oxygen features for LCO and CuO. Additionally, LCO shows intense lanthanum features and also a carbon feature, presumably arising from adsorbates at the surface. **c**,**d**: XPS of the Cu 2p region of LCO and CuO, respectively. The characteristic satellites ($3d^9$) next to the main lines ($3d^{10}$ L^{-1}) confirm the formal +2 oxidation state of Cu in LCO and CuO. Source data are provided as a Source Data file.

Supp. Info 5. Experimental Parameters

	LCO	LCO (wide EW)	CuO
E_{ph} [eV]	770	770	720
E_c AE [eV]	508	508	509
ΔE AE [eV]	0.5	1.0	0.5
E_c PE [eV]	234.5	234.5	185
ΔE PE [eV]	0.2	0.2	0.15
Lens mode AE	'Ang56_4pc'	'Ang50_7pc'	'Ang56_4pc'
Counts True map	177k	314k	216k
Counts Acc. map	579k	1380k	535k
Acc./True ratio	3.3	4.4	2.5
Record time	7h30min	12 h	4h30min*

Supplementary Table 1 Experimental parameters of coincidence measurements for LCO, LCO with wide energy window (EW) and CuO: -From top to bottom- E_{ph}: photon energy; E_c AE: centre energy of Auger electron spectrometer; ΔE AE: energy resolution of Auger electron spectrometer; E_c PE: centre energy of photoelectron spectrometer; ΔE AE: energy resolution of photoelectron spectrometer; Lens mode PE is 'Ang56_4pc' in all measurements; total count numbers of true and accidental coincidence maps; ratios of the total count numbers; * CuO was measured at an effective photon pulse repetition rate of 2.5 MHz leading to an effective measurement time of 9 hour.

Supplementary References

[1] Tanuma, S., Powell, C.J., Penn, D.R.: Calculation of electron inelastic mean free paths (imfps) vii. reliability of the tpp-2m imfp predictive equation. Surface and interface analysis **35**(3), 268–275 (2003)

[2] Jensen, E., Bartynski, R.A., Hulbert, S.L., Johnson, E.D.: Auger photoelectron coincidence spectroscopy using synchrotron radiation. Rev. Sci. Instrum. **63**, 3013–3026 (1992)

[3] Kühn, D., Sinha, S., Johansson, F.O., Ovsyannikov, R., Lindblad, A., Föhlisch, A., Mårtensson, N.: Enhanced surface determination beyond photoemission via auger photoelectron coincidence spectroscopy. The Journal of Physical Chemistry Letters **15**, 8161–8166 (2024)

[4] Pickett, W.E., Krakauer, H., Papaconstantopoulos, D., Boyer, L.: Evidence of conventional superconductivity in la-ba-cu-o compounds. Physical Review B **35**(13), 7252 (1987)

[5] Ching, W., Xu, Y.-N., Wong, K.: Ground-state and optical properties of cu_2o and cuo crystals. Physical Review B **40**(11), 7684 (1989)

[6] Anisimov, V.I., Zaanen, J., Andersen, O.K.: Band theory and mott insulators: Hubbard u instead of stoner i. Physical Review B **44**(3), 943 (1991)

[7] Mahana, D., Mauraya, A.K., Singh, P., Muthusamy, S.K.: Evolution of cuo thin films through thermal oxidation of cu films prepared by physical vapour deposition techniques. Solid State Communications **366**, 115152 (2023)
